# Short-Term Isoflavone Intervention in the Treatment of Severe Vasomotor Symptoms after Surgical Menopause: A Case Report and Literature Review

**DOI:** 10.1155/2015/962740

**Published:** 2015-10-29

**Authors:** Supanimit Teekachunhatean, Natnita Mattawanon, Surapan Khunamornpong

**Affiliations:** ^1^Department of Pharmacology, Faculty of Medicine, Chiang Mai University, Chiang Mai 50200, Thailand; ^2^Center of Thai Traditional and Complementary Medicine, Faculty of Medicine, Chiang Mai University, Chiang Mai 50200, Thailand; ^3^Department of Obstetrics and Gynecology, Faculty of Medicine, Chiang Mai University, Chiang Mai 50200, Thailand; ^4^Department of Pathology, Faculty of Medicine, Chiang Mai University, Chiang Mai 50200, Thailand

## Abstract

Isoflavones are soy phytoestrogens that potentially exert various favorable effects in postmenopausal women, for example, alleviating vasomotor episodes, attenuating bone loss, and stimulating vaginal epithelial maturation. There has, however, been lack of consensus regarding those therapeutic effects. Most clinical studies of isoflavones have been conducted with women who had undergone natural menopause, but not those who had undergone surgical menopause. This study reports on a 51-year-old woman who presented with severe vasomotor episodes after undergoing a hysterectomy and a bilateral oophorectomy due to hypermenorrhea secondary to myoma uteri. She refused hormone therapy due to fear of adverse drug reactions so was treated with oral soy isoflavones (two capsules twice daily, equivalent to at least 100 mg daily dose) for 8 weeks. The number and severity of hot flushes and her menopause-specific quality of life dramatically improved from baseline values. The serum bone resorption marker (beta C-telopeptide) decreased markedly, while vaginal epithelial maturation improved slightly, suggesting the potential of isoflavones in attenuating bone loss and stimulating vaginal maturation. The intervention did not adversely affect the hormonal profile (FSH, LH, and estradiol) and liver or renal functions. Thus, isoflavones could be an option for women experiencing severe vasomotor episodes after surgical menopause.

## 1. Introduction

Surgical menopause is defined as the cessation of menstruation in a premenopausal woman who has undergone a bilateral oophorectomy (surgical removal of the ovaries) [1, 2]. The result is an abrupt menopause in which women often experience more severe menopausal symptoms than those who have undergone menopause naturally [2]. These include vasomotor episodes (hot flushes, hot flashes, and night sweats), sexual dysfunction, vaginal dryness, depression, and cardiac symptoms. Furthermore, premature menopause resulting from a bilateral oophorectomy is associated with earlier onset of osteoporosis and coronary heart disease [2]. Estrogen only, that is, estrogen without progestogen, is a regimen of choice for alleviating vasomotor and/or genital atrophic symptoms as well as for attenuating bone loss in women who have undergone surgical menopause [3]. However, the long-term use of estrogen in this population significantly increases the risk of stroke [4]. Additionally, estrogen is not considered a suitable choice for candidates with contraindications such as known or suspected estrogen-dependent neoplasia, active or recent stroke, and known hypersensitivity to estrogen [5]. Concern over the possible adverse reactions of estrogen therapy has led many women to look for natural alternatives that offer benefits comparable to those of estrogen but without the risk of serious complications.

**Figure 1:**
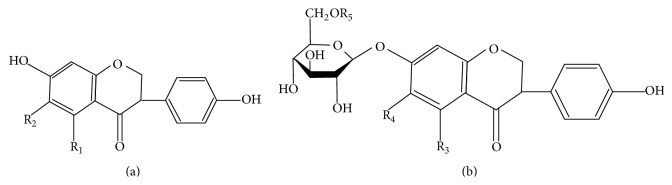
Chemical structures of isoflavones. (a) Unconjugated aglycones. (b) Respective glycoside conjugates. Each with different side chains attached to R1–R5 [7].

Soy isoflavones, the most common group of phytoestrogens, have a chemical structure that resembles mammalian estrogen (estrodiol-17*β*) [6]. Isoflavones can bind to estrogen receptors with a higher affinity for estrogen receptor beta (ER-*β*, found in various tissues and organs such as brain, bone, and arteries) compared to estrogen receptor alpha (ER-*α*, expressed in a wide range of organs such as reproductive organs, that is, the uterus, ovaries, and breast) [6, 7]. In fermented soy foods (e.g., miso and tempeh), isoflavones appear mainly as unconjugated aglycone forms, whereas in nonfermented soy-based products (e.g., soybeans, soy flour, tofu, and soymilk), their respective glycoside conjugates dominate [8, 9] (Figure 1).

Several lines of evidence suggest that isoflavones exert various favorable effects on postmenopausal women such as alleviating vasomotor episodes [10–13], attenuating bone loss [14–18], and maintaining the lining of the vagina (thus reducing vaginal irritation and dryness) [19–22]. With regard to safety, isoflavone intervention exhibits an acceptable safety profile on endometrial morphology (in women who have not undergone hysterectomy), mammography, hormonal levels (follicle-stimulating hormone (FSH), luteinizing hormone (LH), and estradiol), blood lipids, and blood coagulation. In addition, isoflavones, unlike conventional hormone therapy, do not appear to increase the risk of breast cancer or heart disease [10, 11, 23, 24]. However, there has been lack of consensus regarding those therapeutic effects, especially in management of menopausal symptoms [25–27] and osteoporosis [16, 28–32]. The conflicting evidence from different studies could result from differences in the types and doses of isoflavones used, characteristics of individual participants (e.g., ethnicity, age, menopausal status, and baseline symptoms), aspects of the study design, selection of measured outcome variables [33], and so forth.

Clinical studies of isoflavones in postmenopausal women, both those with positive and with negative results, have been extensively conducted in women who have undergone natural menopause. Evidence regarding therapeutic outcomes in women, including women in the Thai population, who have undergone surgical menopause is still lacking. This report describes the highly favorable therapeutic outcomes of isoflavone intervention, in particular, its ability to alleviate the number and severity of vasomotor episodes, including the effects on bone turnover and vaginal cytology as well as safety profiles, in a woman suffering from severe vasomotor episodes after surgical menopause.

## 2. Case Report

A 51-year-old Thai woman presented with severe vasomotor episodes manifested as sensations of warmth (hot flushes) felt on the chest, neck, and face and associated with perspiration and palpitations. The symptoms started shortly after she underwent a hysterectomy and a bilateral oophorectomy due to hypermenorrhea secondary to myoma uteri. In the first five months after surgery, the number of hot flushes gradually increased to approximately six per day; the flushes often disturbed her sleep at night. She refused hormone therapy due to fear of adverse drug reactions.

Physical examination found a generally normal appearance with a body weight of 48 kg, a height of 1.55 m, and a body mass index of 19.98 kg/m^2^. Her body temperature was normal; blood pressure and heart rate were 120/91 mm Hg and 76 bpm, respectively. Other results of the physical examination, including a pelvic examination, were within normal limits. Baseline data (i.e., number and severity of hot flushes, rating of menopause-specific quality of life, hormonal profile, serum bone marker level, and vaginal cytology assessment from Papanicolaou stained smear) are shown in Table 1. At baseline and during treatment, fasting morning blood samples were collected for quantification of serum bone marker levels (beta C-telopeptide or *β*-CTx) and other blood tests. Vaginal smears for cytological examination were collected with a vaginal spatula from the posterior aspect of the upper third of the vaginal wall.

The patient was treated with the Flava soy brand of soy isoflavone capsules (each capsule containing not less than 25 mg isoflavones, manufactured by Thai Herbal Products Co., Ltd., Thailand). The oral isoflavone preparation was prescribed as two capsules twice daily, after breakfast and dinner for a period of 8 weeks. Therapeutic outcomes and adherence to isoflavone treatment are shown in Table 1. The adherence to isoflavone treatment (drug compliance) was found to be more than 90% during the entire treatment period. The mean number of hot flushes per day, the severity of hot flushes, and the menopause-specific quality of life questionnaire scores improved slightly from their respective baseline values after a four-week intervention, but a much greater improvement was observed at the end of an eight-week intervention. After eight weeks, the serum bone resorption marker (*β*-CTx) had decreased considerably, while the vaginal maturation value (determined based on the ratio of superficial, intermediate, and parabasal cells for each specimen) improved slightly. Serum levels of FSH, LH, and estradiol changed minimally from their baseline values, as did blood tests used to assess liver function (aspartate aminotransferase, alanine aminotransferase, alkaline phosphatase, bilirubin, albumin, and globulin) and renal function (creatinine).

The patient was greatly satisfied with the results of the eight-week isoflavone intervention: her hot flushes had disappeared almost completely, so she voluntarily discontinued the treatment at that time. At follow-up four weeks after discontinuation, the patient continued to report an alleviation of hot flushes with the mean number of hot flushes per day found to be very low (0.36 ± 0.49), corresponding to “mild” severity.

## 3. Discussion

The findings from this study found that a twice-daily oral administration of isoflavones (at least 100 mg/day) for eight weeks was very successful in alleviating vasomotor symptoms without affecting endogenous estradiol levels in a woman who had undergone surgical menopause. This finding is consistent with the previously reported results showing that approximately the same daily dose (and dosing frequency) of isoflavone intervention was very effective in reducing hot flushes after natural menopause [11, 34, 35]. Lower daily doses (e.g., 30–90 mg of total isoflavones or 54 mg of pure genistein aglycone) have also been shown to be effective in natural menopausal women [10, 12, 19, 26, 36–38]. It is well known that a significant reduction in vasomotor symptoms is related not only to daily dose of isoflavones but also to the number of baseline hot flushes per day [11, 39, 40]. It has been demonstrated that isoflavone intervention in a candidate with baseline symptoms of more than five flushes per day, such as the one presented in this case report, could be anticipated to achieve a positive therapeutic outcome [12]. It is noteworthy that in this case report vasomotor symptoms declined significantly after only eight weeks of treatment which is contradictory to findings from other studies which have reported significant favorable outcomes following longer duration treatments of 3–12 months [10–12]. This report, therefore, suggests that eight weeks of treatment may be sufficient to judge whether or not isoflavone intervention is effective in alleviating vasomotor symptoms after surgical menopause. Although the mechanism underlying the ability of isoflavones to relieve vasomotor symptoms is not yet fully understood, interaction with the physiological neuroendocrine mechanism of body temperature regulation, in part mediated via ER-*β* in the hypothalamus, is thought to be involved [41].

Since the terminal half-life of isoflavones following an oral administration of soy extract capsule in Thai postmenopausal women has been reported to be relatively short (approximately 6.8–8.3 hours) [42], the sustained therapeutic effect after treatment discontinuation therefore was not anticipated. However, at four weeks after discontinuation, the significant alleviation of vasomotor symptoms in this case could still be observed. There are at least two possibilities for such unusual long-term relief of vasomotor symptoms. First, vasomotor symptoms (especially, early severe profile) can simply decline over time after menopause [43, 44], in part due to time-related central nervous system (CNS) adaptations via different neurochemical pathways in an effort to restore normal temperature regulation [45]. Second, as the patient could have concluded that isoflavone treatment had resulted in the alleviation of vasomotor symptoms, she might have increased her dietary intake of other soy-based products after treatment discontinuation which might be therapeutically equivalent to taking isoflavone capsules.

Bone turnover markers are compounds that give highly relevant information on rates of bone resorption and formation. They also provide information about the effectiveness of osteoporosis treatment, in spite of not being useful alone to estimate bone loss [46]. It has been demonstrated that isoflavone intervention can prevent bone resorption, but its benefits for bone formation are not significant [29, 30, 47–50]. Bone resorption markers, especially serum C-terminal telopeptide of type I collagen (CTx) and its degradation fragments that contain the beta-isomerized octapeptide EKAHD-beta-GGR (*β*-CTx), are therefore better indicators than bone formation markers for assessing the response to intervention in postmenopausal women [51, 52]. The fasting morning blood samples were collected for quantification of serum *β*-CTx levels to avoid being confounded by circadian variation and food intake [52]. It is not surprising that serum *β*-CTx in this case considerably decreased from baseline after an eight-week isoflavone intervention as other studies have shown that isoflavones can significantly decrease various bone resorption markers (i.e., CTx, pyridinoline, and deoxypyridinoline) [47–49, 53]. In fact, this desirable effect may be observed in as early as three weeks following the intervention [47]. It is possible that a decline in serum *β*-CTx such as that found in this study might be able to predict subsequent improvement in the lumbar spine or femoral bone mineral density (if patient continues taking isoflavone capsules for one or two years) [51]. The favorable effect on bone is thought to be mainly mediated via a weak estrogen agonistic effect of isoflavones on ER-*β*. Other potential mechanisms of isoflavone action involve alteration in the receptor activator of the nuclear factor-kB ligand- (RANKL-) osteoprotegerin (OPG) pathway, inhibition of nuclear factor-kB activation, inhibition of production of tumor necrotic factor alpha (TNF-*α*), interleukin-1, interleukin-6, and so forth [16].

Estrogen deficiency, after either natural or surgical menopause, can subsequently lead to the pathophysiologic changes associated with urogenital atrophy characterized by vaginal dryness, irritation, and dyspareunia. Estrogen therapy significantly stimulates vaginal epithelial maturation resulting in improvement in vaginal maturation value while relieving symptoms of vulvovaginal atrophy [54]. Similarly, the eight-week isoflavone intervention in this case stimulated vaginal epithelial cell maturation from parabasal to intermediate type, indicating that isoflavones have an estrogenic property. However, this change appears to be different from the effect of conjugated estrogen which stimulates maturation from parabasal to superficial type, suggesting that the estrogenic effect of isoflavones is weaker than estrogen, and hence has no estrogenic effect (negative feedback) on the pituitary gland (i.e., no significant reduction in FSH level) as was observed in this case [20]. The present findings are comparable to the results of other studies demonstrating favorable effects of 61.3 and 165 mg/day doses of isoflavones on vaginal epithelial maturation with no effect on FSH levels after a four-week intervention [20, 21]. The mild improvement in vaginal maturation could possibly be related to a slight improvement in the sexual domain as assessed by the menopause-specific quality of life questionnaire which is used to investigate sexual and vaginal symptoms in postmenopausal women [55]. However, the positive effects on vaginal maturation appear to contradict findings from other clinical studies which found no significant stimulation in vaginal epithelial maturation [10, 11, 56]. In addition to the previously mentioned factors potentially contributing to the conflicting results, the number and distribution of estrogen receptors (especially ER-*α*) as well as their response to isoflavone binding could possibly contribute to an individual variability [56].

Given that the clinical benefits of isoflavone intervention in postmenopausal women remain controversial and its effectiveness in women who have undergone surgical menopause needs to be further elucidated, the successful therapeutic outcome in this case seems to result primarily from several factors such as the relative high-dose of isoflavone extract (at least 100 mg/day), adequate duration of treatment (eight weeks), suitable baseline symptoms (more than five flushes per day), and, perhaps, Thai ethnicity. These findings can serve as a guide for designing future randomized controlled studies in Thai and other populations.

It has been previously shown in Thai postmenopausal women that coadministration of vitamin D and calcium (which are generally recommended in postmenopausal women) and isoflavone extract does not adversely affect the oral bioavailability of isoflavones [42]. In addition, pre- or synbiotic supplementation has been demonstrated to significantly enhance the oral bioavailability of isoflavones [57, 58], so administration of isoflavones plus vitamin D/calcium and pre- or synbiotics should be considered another option to maximize clinical benefits in this target group.

## 4. Conclusions

A twice-daily oral administration of isoflavones (at least 100 mg/day) for eight weeks significantly alleviated vasomotor symptoms, decreased bone resorption marker (*β*-CTx), and resulted in minor improvement in vaginal epithelial maturation in a woman who had experienced severe vasomotor symptoms after surgical menopause. The intervention did not adversely affect the hormonal profile and liver or renal function. Isoflavones can, therefore, be considered an attractive alternative option in this target population.

## Figures and Tables

**Table 1:** Variables measured at weeks 0 (baseline), 4, and 8.

Variable	Week 0	Week 4	Week 8
Mean number of hot flushes per day^1^ [59, 60]	6.00 ± 0.82	4.04 ± 1.00	0.82 ± 1.49
Severity of hot flushes^2^ [59, 60]	Moderate	Moderate	Mild
Menopause-specific quality of life questionnaire score^3^ [55, 61]			
Vasomotor domain	16	15	4
Psychosocial domain	13	15	9
Physical domain	44	51	41
Sexual domain	7	5	3
Total	80	86	57
Serum bone marker			
Beta C-telopeptide; *β*-CTx (ng/mL)	1.28	ND	0.54
Proportion of cell types in vaginal smear			
Parabasal cells (%)	100	ND	70.8
Intermediate cells (%)	0	ND	20.4
Superficial cells (%)	0	ND	1.2
Vaginal maturation value (VMV)^4^	0	ND	11.4
Hormonal profile			
Follicle-stimulating hormone (FSH) (mIU/mL)	118.70	ND	111.30
Luteinizing hormone (LH) (mIU/mL)	87.87	ND	71.77
Estradiol (pg/mL)	<5.00	ND	<5.00
Adherence to isoflavone treatment^5^ (%)	ND	91.96%	92.86%

ND: not determined. ^1^The number of hot flushes was self-reported by the patient once daily; values represent the average number in a given week. ^2^The severity of hot flushes was categorized according to the mean number of hot flushes in a given week. ^3^Higher scores indicate poorer quality of life. ^4^VMV has a range of 0–100, derived from a 300-cell count and calculated using the equation (1 × percentage of superficial cells) + (0.5 × percentage of intermediate cells) + (0 × percentage of parabasal cells).  ^5^Adherence to treatment was determined from consumption of the isoflavone capsules which had been prescribed at the previous visit.

## References

[1] Gallicchio L., Whiteman M. K., Tomic D., Miller K. P., Langenberg P., Flaws J. A. (2006). Type of menopause, patterns of hormone therapy use, and hot flashes. *Fertility and Sterility*.

[2] Hendrix S. L. (2005). Bilateral oophorectomy and premature menopause. *The American Journal of Medicine*.

[3] Nelson H. D. (2008). Menopause. *The Lancet*.

[4] MacLennan A. H., Sturdee D. W. (2004). WHI, WHI, WHI?. *Climacteric*.

[5] Windler E., Stute P., Ortmann O., Mueck A. O. (2015). Is postmenopausal hormone replacement therapy suitable after a cardio- or cerebrovascular event?. *Archives of Gynecology and Obstetrics*.

[6] Setchell K. D. R. (1998). Phytoestrogens: the biochemistry, physiology, and implications for human health of soy isoflavones. *The American Journal of Clinical Nutrition*.

[7] Teekachunhatean S., Hanprasertpong N., Teekachunhatean T. (2013). Factors affecting isoflavone content in soybean seeds grown in Thailand. *International Journal of Agronomy*.

[8] Murphy P. A., Barua K., Hauck C. C. (2002). Solvent extraction selection in the determination of isoflavones in soy foods. *Journal of Chromatography B: Analytical Technologies in the Biomedical and Life Sciences*.

[9] Wang H.-J., Murphy P. A. (1994). Isoflavone content in commercial soybean foods. *Journal of Agricultural and Food Chemistry*.

[10] D'Anna R., Cannata M. L., Marini H. (2009). Effects of the phytoestrogen genistein on hot flushes, endometrium, and vaginal epithelium in postmenopausal women: a 2-year randomized, double-blind, placebo-controlled study. *Menopause*.

[11] Nahas E. A. P., Nahas-Neto J., Orsatti F. L., Carvalho E. P., Oliveira M. L. C. S., Dias R. (2007). Efficacy and safety of a soy isoflavone extract in postmenopausal women: a randomized, double-blind, and placebo-controlled study. *Maturitas*.

[12] Crisafulli A., Marini H., Bitto A. (2004). Effects of genistein on hot flushes in early postmenopausal women: a randomized, double-blind EPT- and placebo-controlled study. *Menopause*.

[13] Chen M. N., Lin C. C., Liu C. F. (2015). Efficacy of phytoestrogens for menopausal symptoms: a meta-analysis and systematic review. *Climacteric*.

[14] Marini H., Minutoli L., Polito F. (2007). Effects of the phytoestrogen genistein on bone metabolism in osteopenic postmenopausal women: a randomized trial. *Annals of Internal Medicine*.

[15] Wong W. W., Lewis R. D., Steinberg F. M. (2009). Soy isoflavone supplementation and bone mineral density in menopausal women: a 2-y multicenter clinical trial. *The American Journal of Clinical Nutrition*.

[16] Atmaca A., Kleerekoper M., Bayraktar M., Kucuk O. (2008). Soy isoflavones in the management of postmenopausal osteoporosis. *Menopause*.

[17] Ma D.-F., Qin L.-Q., Wang P.-Y., Katoh R. (2008). Soy isoflavone intake increases bone mineral density in the spine of menopausal women: meta-analysis of randomized controlled trials. *Clinical Nutrition*.

[18] Taku K., Melby M. K., Takebayashi J. (2010). Effect of soy isoflavone extract supplements on bone mineral density in menopausal women: meta-analysis of randomized controlled trials. *Asia Pacific Journal of Clinical Nutrition*.

[19] Carmignani L. O., Pedro A. O., Costa-Paiva L. H., Pinto-Neto A. M. (2010). The effect of dietary soy supplementation compared to estrogen and placebo on menopausal symptoms: a randomized controlled trial. *Maturitas*.

[20] Uesugi T., Toda T., Okuhira T., Chen J.-T. (2003). Evidence of estrogenic effect by the three-month-intervention of isoflavone on vaginal maturation and bone metabolism in early postmenopausal women. *Endocrine Journal*.

[21] Baird D. D., Umbach D. M., Lansdell L. (1995). Dietary intervention study to assess estrogenicity of dietary soy among postmenopausal women. *The Journal of Clinical Endocrinology and Metabolism*.

[22] Ghazanfarpour M., Latifnejad Roudsari R., Treglia G., Sadeghi R. (2015). Topical administration of isoflavones for treatment of vaginal symptoms in postmenopausal women: a systematic review of randomised controlled trials. *Journal of Obstetrics and Gynaecology*.

[23] McCarty M. F. (2006). Isoflavones made simple—genistein's agonist activity for the beta-type estrogen receptor mediates their health benefits. *Medical Hypotheses*.

[24] D'Adamo C. R., Sahin A. (2014). Soy foods and supplementation: a review of commonly perceived health benefits and risks. *Alternative Therapies in Health and Medicine*.

[25] Knight D. C., Howes J. B., Eden J. A., Howes L. G. (2001). Effects on menopausal symptoms and acceptability of isoflavone-containing soy powder dietary supplementation. *Climacteric*.

[26] Khaodhiar L., Ricciotti H. A., Li L. (2008). Daidzein-rich isoflavone aglycones are potentially effective in reducing hot flashes in menopausal women. *Menopause*.

[27] Penotti M., Fabio E., Modena A. B., Rinaldi M., Omodei U., Viganó P. (2003). Effect of soy-derived isoflavones on hot flushes, endometrial thickness, and the pulsatility index of the uterine and cerebral arteries. *Fertility and Sterility*.

[28] Liu J., Ho S. C., Su Y.-X., Chen W.-Q., Zhang C.-X., Chen Y.-M. (2009). Effect of long-term intervention of soy isoflavones on bone mineral density in women: a meta-analysis of randomized controlled trials. *Bone*.

[29] Lydeking-Olsen E., Beck-Jensen J.-E., Setchell K. D. R., Holm-Jensen T. (2004). Soymilk or progesterone for prevention of bone loss—a 2 year randomized, placebo-controlled trial. *European Journal of Nutrition*.

[30] Nikander E., Metsä-Heikkilä M., Ylikorkala O., Tiitinen A. (2004). Effects of phytoestrogens on bone turnover in postmenopausal women with a history of breast cancer. *The Journal of Clinical Endocrinology and Metabolism*.

[31] Brink E., Coxam V., Robins S., Wahala K., Cassidy A., Branca F. (2008). Long-term consumption of isoflavone-enriched foods does not affect bone mineral density, bone metabolism, or hormonal status in early postmenopausal women: a randomized, double-blind, placebo controlled study. *The American Journal of Clinical Nutrition*.

[32] Albertazzi P., Steel S. A., Bottazzi M. (2005). Effect of pure genistein on bone markers and hot flushes. *Climacteric*.

[33] Lagari V. S., Levis S. (2010). Phytoestrogens and bone health. *Current Opinion in Endocrinology, Diabetes and Obesity*.

[34] Chedraui P., San Miguel G., Schwager G. (2011). The effect of soy-derived isoflavones over hot flushes, menopausal symptoms and mood in climacteric women with increased body mass index. *Gynecological Endocrinology*.

[35] Crawford S. L., Jackson E. A., Churchill L., Lampe J. W., Leung K., Ockene J. K. (2013). Impact of dose, frequency of administration, and equol production on efficacy of isoflavones for menopausal hot flashes: a pilot randomized trial. *Menopause*.

[36] Elkind-Hirsch K. (2001). Effect of dietary phytoestrogens on hot flushes: can soy-based proteins substitute for traditional estrogen replacement therapy?. *Menopause*.

[37] Messina M., Messina V. (2003). Provisional recommended soy protein and isoflavone intakes for healthy adults: rationale. *Nutrition Today*.

[38] Branca F., Lorenzetti S. (2005). Health effects of phytoestrogens. *Forum of Nutrition*.

[39] Messina M., Hughes C. (2003). Efficacy of soyfoods and soybean isoflavone supplements for alleviating menopausal symptoms is positively related to initial hot flush frequency. *Journal of Medicinal Food*.

[40] Howes L. G., Howes J. B., Knight D. C. (2006). Isoflavone therapy for menopausal flushes: a systematic review and meta-analysis. *Maturitas*.

[41] Bu L.-H., Lephart E. D. (2005). Effects of dietary phytoestrogens on core body temperature during the estrous cycle and pregnancy. *Brain Research Bulletin*.

[42] Teekachunhatean S., Pongnad P., Rojanasthein N., Manorot M., Sangdee C. (2011). Effects of vitamin D plus calcium supplements on pharmacokinetics of isoflavones in Thai postmenopausal women. *Evidence-Based Complementary and Alternative Medicine*.

[43] Mishra G. D., Kuh D. (2012). Health symptoms during midlife in relation to menopausal transition: British prospective cohort study. *British Medical Journal*.

[44] Mishra G. D., Dobson A. J. (2012). Using longitudinal profiles to characterize women's symptoms through midlife: results from a large prospective study. *Menopause*.

[45] Deecher D. C., Dorries K. (2007). Understanding the pathophysiology of vasomotor symptoms (hot flushes and night sweats) that occur in perimenopause, menopause, and postmenopause life stages. *Archives of Women's Mental Health*.

[46] Lee J., Vasikaran S. (2012). Current recommendations for laboratory testing and use of bone turnover markers in management of osteoporosis. *Annals of Laboratory Medicine*.

[47] Sharif P. S., Nikfar S., Abdollahi M. (2011). Prevention of bone resorption by intake of phytoestrogens in postmenopausal women: a meta-analysis. *Age*.

[48] Ma D.-F., Qin L.-Q., Wang P.-Y., Katoh R. (2008). Soy isoflavone intake inhibits bone resorption and stimulates bone formation in menopausal women: meta-analysis of randomized controlled trials. *European Journal of Clinical Nutrition*.

[49] Marini H., Bitto A., Altavilla D. (2008). Breast safety and efficacy of genistein aglycone for postmenopausal bone loss: a follow-up study. *Journal of Clinical Endocrinology and Metabolism*.

[50] Taku K., Melby M. K., Kurzer M. S., Mizuno S., Watanabe S., Ishimi Y. (2010). Effects of soy isoflavone supplements on bone turnover markers in menopausal women: systematic review and meta-analysis of randomized controlled trials. *Bone*.

[51] Chailurkit L.-O., Ongphiphadhanakul B., Piaseu N., Saetung S., Rajatanavin R. (2001). Biochemical markers of bone turnover and response of bone mineral density to intervention in early postmenopausal women: an experience in a clinical laboratory. *Clinical Chemistry*.

[52] Wheater G., Elshahaly M., Tuck S. P., Datta H. K., van Laar J. M. (2013). The clinical utility of bone marker measurements in osteoporosis. *Journal of Translational Medicine*.

[53] Bevilacqua M., Righini V., Certan D., Gandolini G., Alemanni M. (2013). Effect of a mixture of calcium, vitamin D, inulin and soy isoflavones on bone metabolism in post-menopausal women: a retrospective analysis. *Aging Clinical and Experimental Research*.

[54] Cardozo L., Bachmann G., McClish D., Fonda D., Birgerson L. (1998). Meta-analysis of estrogen therapy in the management of urogenital atrophy in postmenopausal women: second report of the Hormones and Urogenital Therapy Committee. *Obstetrics and Gynecology*.

[55] Hilditch J. R., Lewis J., Peter A. (1996). A menopause-specific quality of life questionnaire: development and psychometric properties. *Maturitas*.

[56] Manonai J., Songchitsomboon S., Chanda K., Hong J. H., Komindr S. (2006). The effect of a soy-rich diet on urogenital atrophy: a randomized, cross-over trial. *Maturitas*.

[57] Teekachunhatean S., Techatoei S., Rojanasthein N., Manorot M., Sangdee C. (2012). Influence of fructooligosaccharide on pharmacokinetics of isoflavones in postmenopausal women. *Evidence-Based Complementary and Alternative Medicine*.

[58] Timan P., Rojanasthien N., Manorot M., Sangdee C., Teekachunhatean S. (2014). Effect of synbiotic fermented milk on oral bioavailability of isoflavones in postmenopausal women. *International Journal of Food Sciences and Nutrition*.

[59] Cao Z. Y. (2005). *Chinese Obstetrics and Gynecology*.

[60] Tao M., Shao H., Li C., Teng Y. (2013). Correlation between the modified Kupperman Index and the Menopause Rating Scale in Chinese women. *Patient Preference and Adherence*.

[61] Pongpatiroj A., Sripramote M., Wanitwanathong G. (2001). Effect of hormone replacement theraphy on the quality of life in postmenopuasal women. *Vajira Medical Journal*.

